# A Direct Assessment of “Obesogenic” Built Environments: Challenges and Recommendations

**DOI:** 10.1155/2011/161574

**Published:** 2011-11-15

**Authors:** Danijela Gasevic, Ina Vukmirovich, Salim Yusuf, Koon Teo, Clara Chow, Gilles Dagenais, Scott A. Lear

**Affiliations:** ^1^Department of Biomedical Physiology and Kinesiology, Simon Fraser University, 2600-515 West Hastings Street, Vancouver, BC, Canada V6B 5K3; ^2^Faculty of Health Sciences, McMaster University, Hamilton Health Sciences Corporation, General Site, McMaster Clinic, 237 Barton Street E., Hamilton, ON, Canada L8L 2X2; ^3^Faculty of Health Sciences, McMaster University, HSC-3U4, 1200 Main Street West, Hamilton, ON, Canada L8N 3Z5; ^4^Sydney Medical School, University of Sydney and The George Institute for Global Health, Level 10 King George V Building, Missenden Road, Camperdown, Sydney NSW 2050, Australia; ^5^Institut Universitaire de Cardiologie et de Pneumologie de Quebec and Université Laval, 2725 Chemin Sainte-Foy, Quebec City, QC, Canada G1V 4G5

## Abstract

This paper outlines the challenges faced during direct built environment (BE) assessments of 42 Canadian communities of various income and urbanization levels. In addition, we recommend options for overcoming such challenges during BE community assessments. Direct BE assessments were performed utilizing two distinct audit methods: (1) modified version of Irvine-Minnesota Inventory in which a paper version of an audit tool was used to assess BE features and (2) a Physical Activity and Nutrition Features audit tool, where the presence and positions of all environmental features of interest were recorded using a Global-Positioning-System (GPS) unit. This paper responds to the call for the need of creators and users of environmental audit tools to share experiences regarding the usability of tools for BE assessments. The outlined BE assessment challenges plus recommendations for overcoming them can help improve and refine the existing audit tools and aid researchers in future assessments of the BE.

## 1. Introduction

Obesity poses a serious threat to the health of populations worldwide [1]. An environment that hinders physical activity and promotes excessive food intake has been implicated as a major contributor to the rising obesity prevalence [2]. Implicit in this is the “built environment” (BE) which refers to the human-made or modified characteristics of the physical environment [3]. The BE can be assessed in a number of ways, and these include the following: *indirect* measures where aspects of the environment are assessed through data gained from secondary sources such as geographic information systems (GIS) or street network data; *direct* measures where BE features are assessed through in-person audits, such as by walking through the targeted environment; and *intermediate* measures which may rely on assessing individual perceptions of neighbourhood characteristics, use of telephone book yellow pages, or aerial photography to identify the variables of interest [4]. The majority of studies investigating BE make use of indirect and intermediate measures [4]; however, indirect measures often lack microscale-level data (such as sidewalk quality, trees presence and incivilities) that are more likely to influence and explain changes in one's daily energy expenditure compared to macroenvironmental features such as land use and features of urban design [5]. Moreover, given that some of the most commonly used indirect methods such as GIS rely on existing and often not regularly updated BE databases (e.g., commercial databases on food stores and recreational facilities), disagreement can arise between what is in the database and what is actually present in the environment [6].

A range of audit tools that is available for direct assessment of BE features [7–20] vary along a number of dimensions such as levels of detail and complexity as well as the level of expertise required to perform the environmental audits [21]. A “gold standard” instrument does not exist [10], and inconsistent approaches for assessing the same built environment features make it difficult to compare results across studies. With the lack of a standardized tool, auditors are faced with challenges during environmental data collection. Identifying and addressing these challenges is necessary to ensure that BE data collection is feasible, accurate, and comparable across settings and studies. Therefore, in this paper we outline challenges commonly faced by and identify some solutions to aid researchers in future assessments of the BE based on our experience in assessing the BE directly in over 40 communities representing a cross-section of environmental heterogeneity.

## 2. Materials and Methods

As part of the Prospective Urban Rural Epidemiology (PURE) study [22], we assessed over nine thousand study participants in Canada for cardiometabolic risk factors, physical activity behaviour, and environmental perceptions (perceived measures). We subsequently performed direct BE assessments of 42 PURE recruitment areas or “communities*”* where 8655 of these assessed participants resided. The communities included 15 Vancouver, 19 Hamilton, and 8 Quebec City areas which were chosen to represent various income levels and comprise urban and rural environments. To conduct direct BE assessments, we used two audit methods: (1) a modified version of the Irvine-Minnesota Inventory (IMI) [7] in which a paper version of an audit tool was used to assess BE features and (2) a Physical Activity and Nutrition Features audit tool, where the presence and positions of all environmental features of interest were recorded using a GPS unit (Garmin models Legend H and HCx, Garmin Ltd., Salem, Oregon). For both methods, each PURE recruitment area, or a “community,” was defined by Forward Sortation Area borders (FSA; regions defined by the first three digits of their postal code). In urban settings, all segments in an FSA were assessed regardless of location of participants. Segments within a 500 m buffer area around FSA were also assessed to take into account the participants living on the border of an FSA. Due to the large expense of some rural areas, however, we limited data collection to the conglomerate of participant 6-digit postal codes within the FSA, plus a 1000 m buffer border to account for the lower density of features present in these rural areas. Each segment (the distance between two intersections) within these communities was audited in order to ensure that all BE features potentially related to active living and obesity were captured. Before conducting the environmental audits, detailed maps of each community were obtained from Google maps [23] and DMTI Spatial Inc. (displayed using ArcGIS 9 geographic information system software, Esri, Redlands, Calif, USA) for modified IMI and GPS/GIS methods, respectively. 

### 2.1. Modified IMI Tool

Prior to commencing the BE assessment of all 42 Canadian communities using the GPS/GIS method, a systematic pilot environmental scan was conducted in two urban income-differing Vancouver communities. For this pilot assessment we utilized the IMI [7], as it has been shown to cover the greatest number and variety of BE variables (162 in total) compared to other available tools at the time [24]. The research assistant performing the audits was first trained using existing IMI training materials then completed a general environmental assessment by driving through the communities. Given the extensive number of BE features covered by the IMI, the purpose of the general assessment was to identify and exclude items covered by the IMI that were not present in communities. Given that only one research assistant was performing the environmental audits, those variables that were subjective in nature (e.g., rating segment attractiveness) were additionally excluded on the basis that subjective ratings are usually related to previous walking experiences of the rater and result in poor interrater reliability [8]. Similarly, front porches, garage doors, and the presence of bars on windows were also excluded, as there were high fences on many properties which restricted clear views of these features. On the other hand, some features were added to the tool such as presence of supermarkets, convenience stores, curbs, or curb cuts (ramps which create a gradient to the curb instead of a step) on places where a sidewalk intersects with an alley. In addition, responses to certain questions were modified to make them more specific. For example, responses to the question “Indicate how many bus stops are on the segment” were as follows: “some/a lot,” “few,” and “none.” Given that we were interested in the number of bus tops present at a particular segment, we modified answer choices to “0”, “1”, “2,” and “3 or more” bus stops. After all modifications, the final audit tool consisted of 125 items (see Table 1 in supplementary material available online at doi: doi:10.1155/2011/161574). Using this tool, we audited a total of 882 segments in one high-income Vancouver community and 750 in another, low-income community. 

### 2.2. Physical Activity and Nutrition Features Audit Tool

Given the volume of data to be collected, we decided to utilize a GPS over the paper version of the Modified IMI tool for recording the presence of BE features in all 42 Canadian communities. We also further customized our modified IMI to meet our goal of quantifying neighbourhood road features, physical activity and nutrition-related facilities, as well as social and educational community facilities, such that we would be able to explore how these BE features relate to the cardiometabolic risk factors and physical activity levels of study participants. Therefore, the final audit tool for assessing the communities included combined features defined by the Irvine-Minnesota Inventory as well as the North American Industry Classification System 2007 [25] (features defined in supplementary Table 2). It evolved based on the experiences gained from the pilot environmental assessments, and it was fine-tuned with the additional input from researchers at the Hamilton and Quebec sites. 

In all 42 communities, trained research assistants recorded the presence and location of neighbourhood BE features (Table 1) with a GPS unit while making notes and keeping track of completed segments on a map of the neighbourhood. In total, there were 3 auditors hired and trained in Vancouver, 3 in Hamilton, and 8 in Quebec (14 in total). We established several training steps to ensure our data were collected consistently between sites. Developed training materials included instructions on installing and using equipment and software and a protocol with detailed written instructions on conducting the assessments, including pre-trip preparation, segment definition instructions for the data collection process, and detailed definitions of each category to be captured. A PowerPoint presentation was also developed to help trainees familiarize themselves with the equipment, to provide pictorial examples of each feature category, and to distinguish between features that may be more difficult to categorize. The PowerPoint also featured a component which showed an example of a walking route and how the features found along the route were to be categorized. A trainer went through the PowerPoint with trainees in-person locally and over the phone in the case of distant centres.

After all the supplied materials were reviewed by trainees, an in-person, hands-on, 3-day training session at each centre was employed in order to make sure all raters understood the training materials and rated environmental features consistently. The first day of the training session involved familiarization with the GPS unit and all its functions/buttons, as well as with the software (Picasa, Google, Mountain View, Calif; Mapsource, Garmin Ltd., Salem, Or; and Copiks PhotoMapper, Kista, Sweden) required for uploading and organizing data. The rest of the training was spent in conducting hands-on assessments in the field with the intention of finding numerous scenarios and a variety of features to practice recording. Topics covered during this time included recording points with the GPS, taking photos (what to take photos of and what to include in the shot), and categorizing features in the neighbourhood (going through feature-dense areas to include as many examples as possible).

Once trained, assessments in densely populated neighbourhoods were carried out by one research assistant working independently and on foot, while in some rural or industrial areas, longer unbroken segments were more easily done by a pair of research assistants in a car—one to drive and the other to perform assessments and call out directions. Irrespective of the transportation used, all streets, paths, and park grounds within each setting were covered by the research team. The average number of segments audited per community was around 1100 for urban FSAs and 570 for rural settings. The total number of segment audited was approximately 14500 in Vancouver, 17000 in Hamilton, and 5000 in Quebec for a total of approximately 36500 segments within the three centres in Canada.

**Figure 1:**
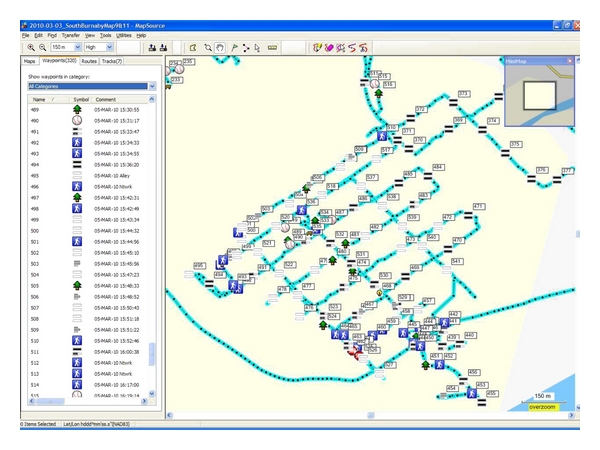
GPS waypoint icons and tracks after uploading into MapSource.

The GPS units used for recording features came with premade waypoint icons and also allowed for a number of custom icons to be added. Each feature of interest therefore had a unique corresponding symbol, and using the included Garmin MapSource software, the icons and their locations were uploaded, viewed, and saved on the computer (Figure 1); these data points were also subsequently mapped in ArcGIS in order to spatially analyze the collected neighbourhood data. Aside from manually recording waypoints, the GPS also gives its user the ability to automatically record location points or “tracks” at preselected intervals. This track data can be used to add location coordinates to photos by matching the date, time, and location of GPS tracks to time stamps of the pictures. During data collection, we used Canon PowerShot digital cameras (Canon Canada, Inc., Mississauga, Ontario) to keep a visual record of neighbourhood features and geotagging software (Copiks PhotoMapper) to later add location information to the images. 

Regardless of the tool/method employed, recording time required per segment depended predominantly on the density of features present on that segment. Using either of the tools, audits required no more time than was needed to walk the length of the segment—up to five minutes for longer segments—where segments lacked features of interest. Segments with a higher density of features required between 8 to 15 minutes (time for taking photos included) to complete using a GPS. The modified version of the IMI required a greater number of features to be assessed compared to the Physical Activity and Nutrition features audit tool, so the assessment time ranged from 5 to 30 minutes depending on the density of environmental features on the segment.

## 3. Results 

While conducting a comprehensive direct assessment of the BE in multiple urban and rural settings, several challenges were encountered which either influenced the shape of our own audit tools or could serve as informing tools for later audits. Namely, taking these challenges into account during the development phase will help researchers modify their audit tools to make assessments smoother and increase interrater consistency. 

One of the challenges experienced by raters at our centres related to categories in which features were recorded such as many/few/none, and solution to this included dichotomizing variables into present/absent or doing counts of the features in order to make the results more objective and give a more accurate representation of the features present. Another challenge was related to timing of assessments: depending on the time of day or season in which audits were conducted, the environments being assessed change. To take into account some of these features, the audit tool should include options to denote seasonality, and timing should be taken into account during the project's planning stages. In combination with tool modifications which will help decrease ambiguity for its users, proper and extensive rater training is also of utmost importance to ensure that the raters completing the audits do so in a thorough and consistent manner. For more specific examples which will better illustrate these and other challenges and solutions, please refer to Table 2.

Despite some of the aforementioned challenges, the majority of environmental features we assessed did not pose any problems. These included features such as residential land uses, presence of public spaces, educational/community/entertainment centres, various institutions, offices and services, sidewalks/sidewalk amenities, bicycle lanes, and measures on the segment to slow down the traffic. Of note, these features tended to be ones that required limited or no subjective assessment and were likely to provide more robust data collection, while the others, presented in Table 2 and discussed in more detail in this paper, are the ones that more training and diligence in assessing is needed for.

## 4. Discussion

We performed direct BE assessments in 42 communities across three urban centres and their surrounding rural areas in different regions of Canada. During this time we came across a number of features that required careful consideration as how to classify them. For example, the direct environmental assessment of some features, such as segment slope, could be influenced by rater's physical fitness, level of fatigue, load that he/she is carrying [26], and walking direction—uphill versus downhill [27]. Consequently, differentiation among categories of steepness might appear challenging in the field. Similar challenges might emerge when assessing the presence of trees. For example, the presence of two large trees on a short segment may be classified differently by multiple raters compared to five or six small trees on a long segment. Similarly, tree type and deciduousness may also affect rater's perception. One procedure that can be employed to help classify ambiguous features is to take photos during data collection to use as later visual reference. Dichotomizing (present/absent) categories such as tree presence, instead of quantifying with nonspecific identifiers (many/some/few), may also help increase inter-rater consistency. Alternatively, and resource allowing, having two or more raters walking and completing assessments together can minimize subjectivity. In addition, working in pairs/groups can allow raters to feel safer in unfamiliar environments, as well as to drive instead of walk in areas with longer, less feature-dense segments (e.g., in rural areas). 

In other cases, features were easy to classify, but they could have better represented the environment with some tool amendments. For example, the IMI prompts for curb cuts to be assessed at intersections, but we encountered a common presence or absence of curb cuts in the middle of street segments where alleys intersect streets. Given that the presence of this feature is especially important for improving sidewalk accessibility and mobility of people using wheelchairs, walking with strollers, or those with limited mobility, it is important to assess the presence of curb cuts not only at street intersections but also at street-alley intersections.

With regards to the method used for data collection, depending on magnitude of the study and the resources available, a paper questionnaire and a GPS unit are both viable options. The use of either requires adequate training, and both have certain advantages and disadvantages (Table 3). However, for the purposes of recording the presence of BE features within multiple large assessment areas, it is our impression that the use of a GPS unit is a more feasible option compared to the paper questionnaire.

Environmental audit tools are essential for conducting direct assessments and enhancing knowledge regarding the built environment features potentially associated with obesity and obesogenic behaviours. Development of high-quality tools that encompass the creation of new measures, the establishment of the reliability and validity of existent ones, and the field testing of these new and existent tools is necessary for further exploration of the association between BE and obesity [28]. Presently, there is considerable variation in environmental measures which is in part due to lack of well-established conceptual models that would present suggestions as to which environmental characteristics should be assessed [29]. Therefore, the development and testing of these conceptual models showing the association between the built environment and obesogenic behaviours has been identified as a priority [30]. Additionally, the identification of shortcomings [31] and the further refinement of existing environmental measures and audit tools should also be emphasized [30]. In order to achieve these objectives, it is important that researchers, both creators and users of environmental audit tools, share their experiences and exchange ideas regarding the usability of tools for direct environmental assessments [31, 32]. Furthermore, it is necessary to ensure the relevancy of environmental measures to populations at high risk for obesogenic behaviours [33]. Consequently, during the development and testing of environmental audits, it is important to engage and receive input from the community [33, 34]. Finally, given that success of a tool depends on the results it produces, results which preferably show little or no variation despite being used by various researchers [35], engaging researchers across various disciplines is also necessary in the process of the development of a valid and reliable BE assessment tool.

## 5. Conclusions

Through the assessment of more than 40 communities in three different Canadian provinces using two distinct methodological approaches, we identified a number of environmental features that posed challenges to classification based on existing audit tools. We have described our suggestions for overcoming such challenges that could help researchers improve and refine the existing audit tools and aid them in future assessments of the BE. The exchange of ideas and fruitful discussion among researchers might help in “harmonizing,” where possible, environmental features from different tools that measure same or similar constructs; this would in turn enhance the comparability of results across studies. Similarly, direct comparability of summary indices such as walkability or traffic safety will be improved when these are calculated from “harmonized” environmental features. Lastly, engagement of the community and researchers from various disciplines will ensure the successful development of valid and reliable audit tools.

## Figures and Tables

**Table 1:** List of the items included in the Physical Activity and Nutrition Features audit tool.

Sidewalks—presence/completion
Shoulders—presence/completion
Paths
Bicycle Lanes
Streetlights
Barriers
Golf courses and country clubs
Skiing facilities
Fitness and recreational sports centres
Bowling centres
Public spaces (green spaces/parks/plazas/squares/courtyards)
Sports fields
Public recreational courts
Outdoor skating rinks
Libraries
Community centres or halls
Places of worship/religious halls
Elementary and secondary schools
Universities, colleges or technical schools/CEGEPS (Quebec)
Entertainment centres
Education/art/nature centres
Mixed use centres
Indoor Shopping centres/malls
Supermarkets and grocery stores
Convenience stores/general stores
Meat or fish/seafood stores/market stores
Fruit and vegetable stores/market stores
Baked goods stores
Confectionary or nut stores
Beer, wine, and liquor stores
Specialty food stores
Full-service restaurants
Limited-service restaurants
(cafés/fast food/pizza places/fast casual restaurants/buffets)
Drinking places (alcoholic beverages)

**Table 2:** Challenges we encountered during the built environment assessment and proposed solutions for those to be overcome.

Environmental feature	Assessment challenges	Proposed modifications
Segment slope	Discerning “steep” from “moderate” slope.	Dichotomize the feature as segment with elevation versus flat segment.
Curb cuts	Curb cuts at alley-street intersections are unaccounted for.	Record presence/absence of curb cuts at both street-alley intersections and street intersections.
Trees	When quantifying trees with nonspecific identifiers (i.e., some/few/none), tree size and type will affect how they are categorized.	Use two absolute categories: trees present versus trees absent. Include park trees/trees in front yards of houses if large enough to give shade to sidewalks and protect from precipitation.
Street parking	Parking rules vary depending on time of day and location.	Include a variety of categories to cover all parking options: for example, free parking on streets, paid parking on streets, time-dependent parking (available only during some parts of the day), no street parking available.
Graffiti and litter	Greater littering behavior noted during holidays. High potential for graffiti tags to be overlooked if not actively sought out.	To increase reliability: for litter avoid assessments during days associated with increased waste production (holidays, community events). For graffiti; training manuals should include probable graffiti locations (e.g., back of traffic signs, post office mail boxes), so that these are not overlooked.
Street lighting	Streetlight coverage may vary between different segments of approximately the same length, resulting in a different “amount” of light available per segment.	Quantify light: capture density of streetlights (number per given length of segment) or, alternatively, use a light meter to measure lighting of streets/sidewalks at night.
Golf course	Covers a large land area on multiple segments, but accessibility is limited to one or two entry points.	If assessing access to a golf course, only entrance location should be noted, not other points of the area the course spans.
Food stores	Classifying food stores which fall into multiple categories (e.g., meat shop that carries a selection of meats as well as other groceries).	Classify according to most prevalent products: for example, if selection of groceries outweighs meat selection, classify as grocery store. Resources allowing, record the types of food available rather than classifying the store itself to more accurately represent neighbourhood food availability.
Seasonal businesses and use of facilities/trails	Existent features that operate seasonally: some may be closed and inconspicuous during time of assessment (e.g., outdoor skating rink during summer assessments or farms selling one type of seasonal produce); others serve multiple functions which are season dependent (e.g., ski hills become hiking trails during summer).	Record all available activities/facilities and businesses (where known) and assign special coding to specify they are season dependent.

**Table 3:** Advantages and disadvantages of assessing built environment features using a paper questionnaire versus GPS device.

	Paper questionnaire	GPS
Advantages	Easy to administer, does not require advanced technical knowledge.	Small, light, portable, convenient, relatively inexpensive, waterproof, allows for quick recording; comes preloaded with dozens of icons; allows for automatic data transfer onto computer, easy geotagging of pictures to show their locations on a map, and easy integration with spatial analysis programs;
Disadvantages	Questionnaires create a heavy load to carry during assessments; manual data entry is time consuming; inconvenient to use when raining.	Requires greater technical knowledge and integration of several computer programs to get data into a form that can be analyzed; possible safety hazard to be carrying expensive equipment alone and in plain sight. Relative disadvantages: acquiring GPS devices outside of major cities may be more difficult and costly; base map (if used) and position accuracy varies depending on location and quality of GPS device used;
